# Note on Therapeutic Value of Petrogen

**Published:** 1905-02

**Authors:** 


### Note on Therapeutic Value of Petrogen.

Every physician will appreciate the value in applied therapeutics of a preparation with which it is possible to obtain the full constitutional effect of a medicament through the medium of local application, for by this method it would be possible to administer such drugs as iodine, creosote, methylsalicylate, and others, without producing disturbances of the gastrointestinal function which often follow their internal administration. In petrogen (notice of which appears on page xviii.), Messrs. John Wyeth & Brother offer a vehicle which is claimed to be non-irritating to the most delicate and inflamed surface and immediately penetrates through the integument or mucous surface, carrying with it the medicament in solution, into the general circulation. It is also asserted as possible with this product to procure not only the therapeutic action of the medication upon the local tissue with which it comes in contact, but to obtain the peculiar systemic effect inherent to the several medicaments.

---

The heirs of Professor Virchow have given $12,500 to be applied toward the prevention of infant mortality in Berlin.—*Med. Age.*

